# Pulmonary Arterial Hypertension-Induced Reproductive Damage: Effects of Combined Physical Training on Testicular and Epididymal Parameters in Rats

**DOI:** 10.3390/biomedicines13020410

**Published:** 2025-02-08

**Authors:** Mírian Quintão Assis, Luciano Bernardes Leite, Luiz Otávio Guimarães-Ervilha, Rui Adão, Emily Correna Carlo Reis, Antônio José Natali, Mariana Machado-Neves

**Affiliations:** 1 Department of General Biology, Federal University of Viçosa, Viçosa 36570-900, MG, Brazil; mirian.assis@ufv.br; 2 Laboratory of Exercise Biology, Federal University of Viçosa, Viçosa 36570-900, MG, Brazil; luciano.leite@ufv.br (L.B.L.); anatali@ufv.br (A.J.N.); 3 Department of Pharmacology and Toxicology, School of Medicine, Complutense University of Madrid, 28040 Madrid, Spain; rdacosta@ucm.es; 4 CIBER Enfermedades Respiratorias (Ciberes), 28029 Madrid, Spain; 5 Instituto de Investigación Sanitaria Gregorio Marañón (IiSGM), 28009 Madrid, Spain; 6 Department of Veterinary Medicine, Federal University of Viçosa, Viçosa 36570-900, MG, Brazil; emily.carlo@ufv.br

**Keywords:** physical exercise, testis, testosterone, epididymis, sperm

## Abstract

**Background/Objectives:** Pulmonary arterial hypertension (PAH) affects the pulmonary vasculature and cardiac function. While its impact on target organs has been extensively studied, little is known about its effects on highly vascularized organs, such as those from the male reproductive system. This study explores the impact of PAH on testis and epididymis, evaluating the potential role of combined exercise training as a non-pharmacological strategy to mitigate alterations in these organs. **Methods:** Male Wistar rats (*n* = 8/group) were assigned to one of three groups: sedentary control, sedentary PAH, and exercise PAH. PAH was induced by monocrotaline administration (60 mg Kg^−1^, i.p). The exercise PAH group underwent three weeks of combined physical training, including treadmill aerobic activity and resistance training on a ladder. Testis and epididymis were analyzed histologically, histomorphometrically, and biochemically for antioxidant activity, oxidative stress markers, and sperm parameters. **Results:** Sedentary PAH animals showed reductions in body and epididymis weight, normal seminiferous tubule percentage, and testicular morphometric parameters. These changes led to disorganized seminiferous tubules and compromised sperm production and sperm count in the testis and epididymis. Combined physical training improved testicular morphometric alterations and increased sperm count in hypertensive animals. **Conclusions:** PAH negatively affects testicular structure and function, leading to low sperm production. Combined physical training mitigated these effects by preserving testicular architecture and improving reproductive parameters, though it appeared less effective for the epididymis. These findings suggest physical training as a potential therapeutic strategy to protect reproductive health in PAH.

## 1. Introduction

Pulmonary arterial hypertension (PAH) is a multifactorial disease with a global prevalence estimated at 192,000 cases in 2021 [1,2,3,4]. The pathophysiology of the disease includes genetic factors, such as the upregulation of DNA methyltransferases and downregulation of vasoprotective genes, and environmental factors, including exposure to toxins (e.g., methamphetamine) and infectious diseases, including AIDS and schistosomiasis [5]. PAH causes remodeling of the pulmonary vasculature by increasing pulmonary arterial pressure, which can promote right ventricular failure and even lead to patient death [6,7]. Therefore, given the severity of the disease, therapeutic alternatives are sought to minimize symptoms and improve the quality of life of affected individuals since there is still no cure for PAH [8]. Among these alternatives, pharmacological and physical practices can improve the clinical picture of the disease [9].

The beneficial effects of physical training on the cardiopulmonary and muscular systems have been evidenced in animal models and human studies [10,11,12,13,14,15]. Clinically relevant improvements from this practice include exercise tolerance, increases in aerobic capacity, muscle strength, and patients’ quality of life [16,17,18]. It is important to emphasize that different exercise programs may cause distinct effects on PAH management. Furthermore, physical activities are safe when well-structured and integrated into comprehensive therapeutic approaches [8,10].

There is still no ideal preclinical model for human PAH. However, monocrotaline, an important inducer of PAH, has often been used in animal experiments, especially in murine models [3,19]. Although the toxic mechanisms of MCT remain unelucidated, this drug damages lung endothelial cells and plays an important role in inflammation and lung injury [20].

Several studies have shown the harmful role of cardiovascular diseases in the male reproductive system, such as low hormone production, tissue disorganization, oxidative stress generation, erectile dysfunction, and low sperm quality [21,22,23]. However, information about PAH and possible reproductive disorders arising from this clinical condition is scarce, as not enough studies have examined the effects of this condition on highly vascularized organs, such as the epididymis and testis, which are particularly susceptible to damage [24]. A pioneering study investigated the impact of PAH on the rat testis and epididymis, revealing that the disease reduces testosterone production, the activity of antioxidant enzymes, and the concentration of nitric oxide, causing disorganization of the testicular and epididymal architecture and impairing the sperm formation and maturation [25,26]. The testis and epididymis play crucial roles in male fertility. The testes are responsible for steroidogenesis and spermatogenesis, producing sperm within the seminiferous tubules [27]. The epididymis, a highly specialized organ adjacent to the testis, provides an ideal environment for sperm maturation, allowing the acquisition of sperm motility and fertile ability and serving as a storage site for mature sperm until ejaculation [28]. Together, these organs ensure the production and functional competence of the male gamete. In men, PAH represents a high risk of erectile dysfunction related to low testosterone concentrations and oxidative stress occurrence, which can lead to endothelial dysfunction. This condition impairs the dilation of the corpus cavernosum arteries, compromising sexual activity [29,30]. Thus, PAH has significant clinical implications for the fertility and sexual health of patients, highlighting the importance of therapeutic strategies that protect reproductive health.

The type, intensity, and duration of physical exercise are essential to determine whether it can positively or negatively impact sexual health. Strenuous physical exercise may generate reactive oxygen species (ROS) in the male reproductive system, with changes in sperm parameters, such as motility, morphology, and DNA integrity, leading to fertility issues. Furthermore, it can negatively modulate the hypothalamic–pituitary–gonadal axis, compromising normal testosterone production. However, when well regulated, physical activity can promote amelioration in reproductive functions, such as improving nitric oxide (NO) bioavailability and endothelial function and decreasing the concentration of oxidative stress and inflammation markers [31,32,33]. Therefore, practicing adequate physical activity is essential for maintaining reproductive health and managing therapeutic strategies to mitigate damages induced by cardiovascular diseases.

Understanding the potential damage caused by PAH to the testis and epididymis is crucial for evaluating the impact on male reproduction, as these organs are directly involved in sperm production and maturity, respectively. Thus, this study aimed to assess the effects of experimental PAH on testicular, epididymal, and sperm parameters. Additionally, we investigated combined physical training as a non-pharmacological therapeutic approach to mitigate the progression of damage to the male reproductive system caused by PAH. PAH detrimentally impacts testicular and epididymal structure and function, leading to impaired sperm production. In turn, combined physical training preserved testicular integrity and improved sperm parameters. The results obtained in this study may contribute to clinical practices in PAH treatment and improve the patient’s quality of life, clarifying the relationship between physical exercise, cardiovascular diseases, and male fertility.

## 2. Materials and Methods

### 2.1. Animals and Experimental Design

This study is part of a broader work that involves the analyses of the effects of combined physical training on morphology and cardiopulmonary function in rats with PAH, induced by MCT, developed at the Department of Physical Education at Universidade Federal de Viçosa (UFV) [34,35]. Wistar rats (age: ~six weeks; body mass: ~200 g) from the Central Animal Facility of the Center for Biological and Health Sciences at UFV were used in the present study.

The animals were housed in polyethylene boxes, with four rats per box, with access to commercial food and filtered water ad libitum. The ambient temperature was maintained at 22 ± 2 °C and a controlled light cycle of 12/12 h light/dark. All experimental procedures were evaluated and approved by the Ethics Committee on the Use of Animals—CEUA UFV (process nº 02/2021). The experiment lasted three weeks, counting from the MCT injection.

**Figure 1:**
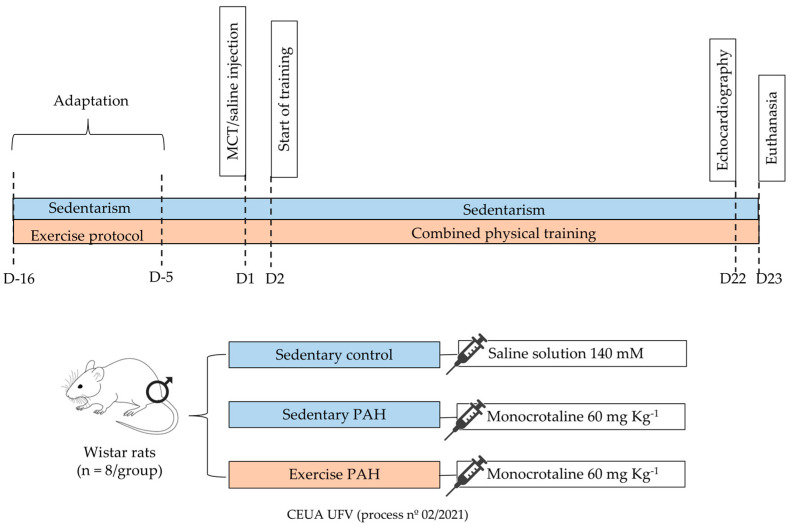
Scheme of experimental design using combined physical training and induction of pulmonary arterial hypertension. The adaptation period began 16 days before the start of the experiment (D16) and lasted 12 days. During the adaptation period (days D16 to D5), animals from the exercise PAH group (*n* = 8/group) were subjected to the combined physical training protocol, while the other sedentary control and sedentary PAH rats (*n* = 8/group) remained in their respective boxes. On experimental day D1, PAH animals received a dose of monocrotaline (60 mg Kg^−1^), while control animals received a saline solution. Twenty-four hours after the injection, rats in the PAH exercise group began combined physical training. On D22, echocardiography was performed, and on D23, the animals were euthanized to collect the testis and epididymis.

The rats were randomly divided into three experimental groups (*n* = 8/group [36]), namely the sedentary control group, containing rats that received a saline solution and were not subjected to combined physical training, the sedentary hypertensive group (sedentary PAH), which included rats that received monocrotaline injection and were not subjected to combined physical training, and the exercise hypertensive group (exercise PAH), composed of rats that received monocrotaline injection and underwent combined physical training (Figure 1).

### 2.2. PAH Induction

PAH was induced with a single intraperitoneal injection of monocrotaline (Sigma-Aldrich, St. Louis, MO, USA) at 60 mg Kg^−1^ body mass, dissolved in 0.5 mL of saline solution (140 mM NaCl; pH 7.4) [17]. This dosage reliably induces severe PAH, aligning with the study’s objective to evaluate the effects under severe conditions. Animals in the sedentary control group received an injection of a saline solution of equal volume.

### 2.3. Combined Physical Training Protocol

The combined moderate-intensity physical training protocol consisted of alternating aerobic training sessions on a motorized treadmill and resistance training sessions on an adapted climbing apparatus, performed five days per week over 21 days. The intensity for each type of training was determined based on pre-evaluations: the maximum running velocity for aerobic training and the maximum carrying load for resistance training, as previously described [34,35,37,38]. Aerobic training sessions lasted 60 min and followed a structured protocol: a 5 min warm-up at 20% of the maximum running velocity, a 50 min training period at 60% of the maximum running velocity, and a 5 min cool-down at 20% of the maximum running velocity. Resistance training sessions consisted of 15 climbs on the climbing apparatus, with a 1 min rest period between each climb. Each session was performed at an intensity equivalent to 60% of the maximum carrying load established during the maximal tests.

### 2.4. Echocardiography

Echocardiographic evaluations were performed on day 22 after the administration of monocrotaline. The animals were anesthetized with isoflurane (1.5%) dissolved in 100% oxygen and delivered at a constant flow rate of 1 L/min (Isoflurane, BioChimico, RJ, Brazil). Images were acquired while the animals were positioned in lateral decubitus. Two-dimensional studies with a high sampling rate of 120 fps in M-mode were conducted using the MyLab™30 ultrasound system (Esaote, Genoa, Italy) with an 11 MHz nominal frequency transducer. Two-dimensional transthoracic echocardiography and M-mode images were obtained at a scanning speed of 200 mm/s, adjusted to the heart rate. Pulmonary artery flow was assessed using pulsed Doppler to measure the acceleration time (AT) and ejection time (ET) in the pulmonary artery, with the AT/ET ratio subsequently calculated. Tricuspid annular plane systolic excursion (TAPSE) was also evaluated and used to indicate right ventricular function. All images were collected following the recommendations of the American Society of Echocardiography and stored for subsequent analysis [39].

### 2.5. Euthanasia and Collection of Biological Material

Animals were euthanized by decapitation on day 23 after monocrotaline injection, as this represents the average survival time of animals with PAH, according to previous studies [18,34,35]. The rats were weighed on the first day of the experiment and on the last day when euthanasia occurred, and the body weight gain of the rats was calculated. The testis and epididymis were removed, dissected, and separated for histomorphometric, enzymatic, and functional analyses. Organs on the left side were separated and fixed in Karnovsky’s fixative solution (4% glutaraldehyde and 4% paraformaldehyde in 0.2 M sodium phosphate buffer/pH 7.3) for 24 h for biometric and microscopic analyses. The right-side organs were frozen for sperm production analysis and/or stored at −80 °C for enzymatic assays.

### 2.6. Organ Biometrics

The testis and epididymis were carefully dissected and weighed using an analytical balance to determine their absolute and relative weights. For relative weight calculation, the absolute weight of each organ was multiplied by 100 and divided by the final body weight of the animals. The testicular parenchyma was weighed after removing the tunica albuginea and subtracting its weight from the total gonadal weight [40].

### 2.7. Histological Processing of Organs

Fixed fragments of testis and the four epididymal regions (initial segment, caput, corpus, and cauda) were dehydrated in an increasing series of ethanol (70%, 80%, 90%, and 100%). They were embedded in 2-hydroxyethyl methacrylate and historesin (Historesin^®^, Leica Microsystems, Nussloch, Germany). Semi-serial sections of 3 μm thickness were obtained using glass knives attached to a rotary microtome (RM 2255; Leica Biosystems, Nussloch, Germany). To avoid repeated analysis of the same histological area, an interval of 30 μm (10 sections) was respected between histological sections evaluated for the testis and 39 μm (13 sections) for the epididymis sections. Slides containing histological sections were stained with toluidine blue + sodium borate (1%), mounted with Entellan^®^ (Merck), and analyzed under an optical microscope (Olympus CX40, Tokyo, Japan).

### 2.8. Normal and Pathological Seminiferous Tubule Count

Histological analysis of the testis was performed using an optical microscope (Olympus CX40, Tokyo, Japan), with 200 seminiferous tubules per animal from each group being analyzed, considering the lumen and seminiferous epithelium containing germ and Sertoli cells. The seminiferous tubules were classified into two types, according to the appearance of histological changes: (i) seminiferous tubules with normal morphology, intact seminiferous epithelium and formed by cells arranged in layers, without the presence of vacuoles, desquamation or germ cells in the lumen; (ii) abnormal seminiferous tubules, with the presence of germ cells and cell debris in the lumen, few layers of germ cells, vacuolization and degeneration of the seminiferous epithelium. The results were expressed as a percentage [41,42].

### 2.9. Epididymal Histopathological Analysis

Histological analysis of the epididymis was performed under an optical microscope (Olympus CX40, Tokyo, Japan) at magnifications of 100, 200, and 400×. This stage involved the observation of tissue characteristics of the organ, such as organization and integrity of the epithelium and lumen of the epididymal duct, as well as connective tissue and its components in the interstitium. The occurrence of germ cells in the lumen, quantity and appearance of spermatozoa in the lumen, change in clear cell morphology, cribriform change in the epithelium, epithelial vacuolization, and presence of inflammatory infiltrates in the interstitium were recorded [43].

### 2.10. Histomorphometry and Testicular Stereology

Ten digital images of random histological fields were captured using a bright-field photomicroscope (Olympus BX-53, Tokyo, Japan) at 10× and 40× for testicular morphometric analyses. The images were analyzed using Image J^®^ v. 1.49 (National Institute of Health, Bethesda, MD, USA) and Image Pro Plus 4.5^®^ (Media Cybernetics, Silver Spring, MD, USA) software.

The volumetric proportion of the components of the testicular parenchyma (seminiferous tubules and intertubule) was performed using the Image J^®^ using a graticule with 266 points incidents on each of the ten digitized images, totaling 2660 points for each animal. Coincident intersections were recorded on components of the tubular compartment, such as epithelium, lumen with sperm, lumen without sperm, and tunica propria, and of the intertubular compartment, such as connective tissue, blood vessels, connective tissue cells. The volumetric proportion of these components (VPx) was determined by the formula VPx (%) = (NP/NPT) × 100, where x = component in question, NP = number of points on the specific component and NPT is the number of total points (2660 per animal) [44]. The volume of each testicular component, expressed in mL, was estimated by multiplying the volumetric proportion of each component by the volume of the parenchyma of a testis, divided by 100. As the density of the mammalian testis is around 1 g mL^−1^, the testis mass, in grams, was considered equal to its volume in mL [45].

The diameter of the seminiferous tubule (µm) was obtained by measuring 30 cross-sections as circular as possible per animal. The cross-sections were in stage VIII of the seminiferous epithelium cycle, characterized by the presence of Sertoli cells, type B spermatogonia, and primary spermatocytes in preleptotene close to the basal lamina, with pachytene spermatocytes occupying the adluminal compartment of the epithelium, followed by rounded spermatids. The elongated spermatids were completely dissociated and adhered only to the luminal edge, with residual bodies in the same region [46,47]. The diameter was determined by two straight lines between the poles of the tubule using the Image ProPlus^®^ program, starting from the tunica propria at one end, passing through the center of the tubule, where the two straight lines met, and ending with the tunica propria at the other end. The tubule diameter was determined as the mean of the two straight lines. In the same sections used to measure the tubular diameter, the height of the seminiferous epithelium was measured from two straight lines drawn in the center of the section, from the base of the epithelium in contact with the tunica propria to the lumen of the tubule, at both poles of the seminiferous tubule. Therefore, the final value found for the epithelium height corresponded to the mean of four opposed measurements. Once we have the tubular diameter and the height of the epithelium, we can calculate the luminal diameter (µm) by subtracting the seminiferous tubule’s diameter from its epithelium’s final height [42].

The volumetric proportion of the intertubule components was performed at 400× magnification, counting 1000 points per animal on connective tissue, lymphatic space, blood vessels, macrophages, and Leydig cells, including coincident points in their nucleus and cytoplasm. The volumetric proportion of the intertubular components was obtained using the formula intertubular compartment (%) = (number of points in the intertubular component/1000 total points) × 100. The formula calculated the volume (mL) of each intertubular component per testis: proportion of the element in the testis (%)/(100 × parenchymal mass of a testis). The volume (mL) of the intertubular elements was calculated based on the percentage of the element in the testis/weight of the parenchyma of 2 testis X 100 [48].

Stereological analyses of Leydig cells were performed at 400× magnification. Leydig nucleus diameter (µm) was determined by averaging 30 nuclei per animal, with five animals per group. The volume occupied by the nucleus was calculated using the average nuclear diameter and the formula 4/3πR^3^, where R = nuclear diameter/2. The volume of the cytoplasm was estimated using the formula percentage of cytoplasm x nuclear volume obtained/nuclear percentage. Thus, the total Leydig cell volume was calculated by summing the nuclear and cytoplasmic volumes [48], and the values were expressed in μm^3^. The number of Leydig cells per testis (NLT) was calculated according to the equation NLT = volume that Leydig cells occupy per testis (μm^3^)/volume of an individual Leydig cell (μm^3^). The volume that Leydig cells occupy per testis (μm^3^) = proportion of Leydig cells in the testis X parenchyma weight of a testis/100 [42].

### 2.11. Analyses of Antioxidant Enzymes and Oxidative Metabolites

Samples of frozen testis, caput, and cauda of the epididymis (100 mg; *n* = 5/group) were separated, homogenized in phosphate buffer (1 mL; pH 7.4), and centrifuged for 10 min at 12,000 rpm, under refrigeration 4 °C. The supernatant was separated for analyses of the activity of the enzymes superoxide dismutase (SOD), catalase (CAT) and glutathione-S-transferase (GST), metabolites of lipid peroxidation, such as malondialdehyde (MDA), nitrosative stress, such as nitric oxide, and Ferric-reducing antioxidant power (FRAP). Such analyses were performed in an ELISA microplate reader (Multiskan SkyHigh, Thermo Fisher Scientific^®^, Waltham, MA, USA).

SOD activity was estimated using the method of Dieterich et al. [49] modified, based on the enzyme’s ability to catalyze the dismutation of superoxide (O^2−^) into hydrogen peroxide (H_2_O_2_) and water. This analysis involves the auto-oxidation of pyrogallol, and the reaction is quantified spectrophotometrically at 320 nm. CAT activity was evaluated by measuring the decomposition rate of H_2_O_2_, quantified spectrophotometrically at 374 nm, as described by Aebi [50]. GST activity was estimated at 340 nm in two intervals (30 s and 90 s), as described by Habig et al. [51] with some modifications and calculated from the rate of formation of the conjugate 1-chloro-2,4-dinitrobenzene (CDNB) with glutathione. The molar extinction coefficient used for CDNB was ε340 = 9.6 mmol L^−1^ cm^−1^. The results of SOD, CAT, and GST were normalized to protein levels in the homogenate and total protein quantification was performed using the Bradford method [52]. Enzyme activity was calculated as units (U) per milligram of protein.

MDA concentration was evaluated using the methodology described by Buege and Aust [53]. The homogenate was mixed with TBARS solution (15% trichloroacetic acid, 0.375% thiobarbituric acid, and 0.25 M HCl). Total MDA concentrations in each sample were determined using a standard curve from known concentrations of 1,1,3,3-tetramethoxypropane. The formation of substances reactive to thiobarbituric acid was monitored at 535 nm. The concentration of nitric oxide was measured using the Griess reagent (1% sulfanilamide and 0.1% naphthyl-ethylene-diamine in 2.5% H_3_PO_4_). The amount of nitrite was determined in the samples and used to indicate nitric oxide synthesis. Nitric oxide from samples was determined using the standard curve with known concentrations of sodium nitrite [54]. Plate reading was performed on absorption at 593 nm. FRAP was determined by the method described by Benzie and Strain [55], involving the iron reduction method. FRAP consists of a colorimetric dosage based on the principle of reduction of the ferric tripyridyltriazine complex (Fe^3+^-TPTZ) to the ferrous form (Fe^2+^-TPTZ) by the antioxidants in a sample, with the development of an intense blue color with maximum absorption at 593 nm.

### 2.12. Daily Testicular Sperm Production, Number of Sperm and Transit Time in the Epididymis

Testicular spermatids resistant to homogenization (stage 19 of spermatogenesis), as well as spermatozoa in the caput/corpus and cauda of the epididymis, were counted [56], following the protocol left testis was decapsulated and weighed and homogenized in 5 mL of NaCl 0.9% containing Triton X-100 0.05%. After a dilution, a sample was transferred to Neubauer chambers (four fields per animal), and the number of mature spermatids was counted. To calculate daily sperm production (DSP), the number of spermatids at stage 19 was divided by 6.1, which is the number of days spermatids are present in the seminiferous epithelium. Likewise, the caput/corpus and cauda portions of the epididymis were cut into small fragments with scissors and homogenized, and sperm were counted as described for the testis. The sperm transit time through the epididymis was determined by dividing the number of sperm in each portion by the DSP [57].

### 2.13. Analyses of Sperm Parameters

Immediately after euthanasia, the cauda region of the animals’ left epididymis was cut three times in a Petri dish containing 500 µL Biggers–Whitten–Whittingham (BWW) medium to allow the release of sperm. This fluid was diluted in 500 μL of Tris-citric-fructose (Tris 3.025 g, citric acid 1.7 g, fructose 1.25 g, and 100 mL of distilled water) heated to 34 °C. Aliquots of fluid were collected to evaluate sperm motility and morphology. To analyze total motility, 10 µL of epididymal fluid was placed between the slide and coverslip, previously heated to 37 °C, and evaluated under 400× magnification under an optical microscope. Cells were classified as mobile or immobile [58], and motility was expressed as a percentage (0–100) [59]. For the analysis of sperm morphology, 50 μL of fluid from the epididymal cauda was fixed in 100 μL of 4% buffered formaldehyde. Two hundred cells were examined under a phase contrast microscope (L-1000B, Bioval, São Paulo, Brazil) at 1000× magnification. Sperm with normal and abnormal morphology (head and tail defects) were counted, with the results expressed as a percentage [60].

### 2.14. Statistical Analyses

The data were tested for normality using the Shapiro–Wilk test. Parametric data were analyzed using one-way analysis of variance (ANOVA), followed by Tukey’s post hoc test for multiple comparisons. Differences were considered significant when *p* < 0.05. Analyses were performed using GraphPad Prism 8.2.1 software (GraphPad Software Inc., SanDiego, CA, USA). The results were expressed as mean ± standard deviation of the mean (S.D.M).

## 3. Results

### 3.1. Validation of PAH Induction

Echocardiography results showed that monocrotaline induced PAH in rats that received its injection. Rats in the sedentary PAH group had a lower pulmonary artery TA/TE ratio and reduced right ventricular systolic function, assessed by TAPSE, compared to animals in the sedentary control and exercise PAH groups (*n* = 6 animals/group) (*p* < 0.0001; Table 1).

### 3.2. Effect of PAH on Testicular and Epididymal Parameters

Animals in the sedentary PAH group had lower final body weight and absolute epididymal weight than those observed in sedentary control rats (*p* < 0.05; Table 1). Initial body weight, body weight gain, absolute weight and relative weight of the testis, and relative weight of epididymis of the animals in the experimental groups did not differ from each other (*p* > 0.05; Table 1).

**Figure 2:**
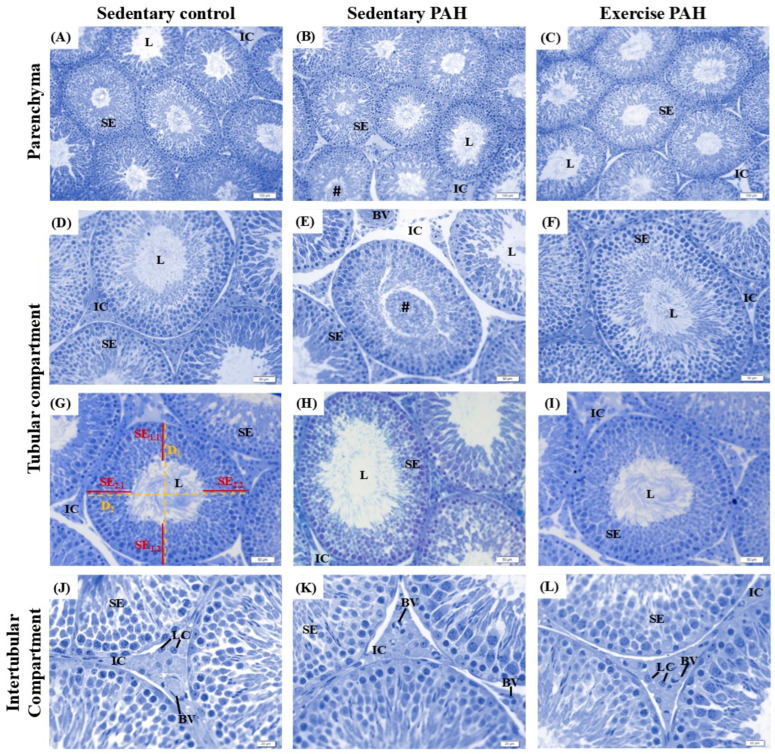
Photomicrographs of the testicular parenchyma of Wistar rats. Sedentary control group (**A**,**D**,**G**,**J**); sedentary PAH group (**B**,**E**,**H**,**K**); exercise PAH group (**C**,**F**,**I**,**L**). Cross-section of seminiferous tubules in stage VIII (**G**–**I**). Dashed yellow lines indicate measurements of tubular diameter. Red lines indicate measurements of seminiferous epithelium height. IC, intertubular compartment; SE, seminiferous epithelium; L, lumen; BV, blood vessel; D = diameter; LC, Leydig cell; # germ cells detached in the lumen. Toluidine blue. Scale bar: (**A**–**C**): 100 µm; (**D**–**I**): 50 µm; (**J**–**L**): 20 µm (*n* = 5 animals/group).

The testis of rats in the sedentary control group presented seminiferous tubules displaying intact epithelium, with regular layers and normal distribution of germ and Sertoli cells, with sperm in the tubular lumen (Figure 2A,D,G). Sedentary HAP rats showed an increase in the percentage of abnormal seminiferous tubules, especially with cells detached from the tubular lumen, compared to that observed in control group animals (*p* < 0.05; Figure 2B,E,H; Table 2).

**Figure 3:**
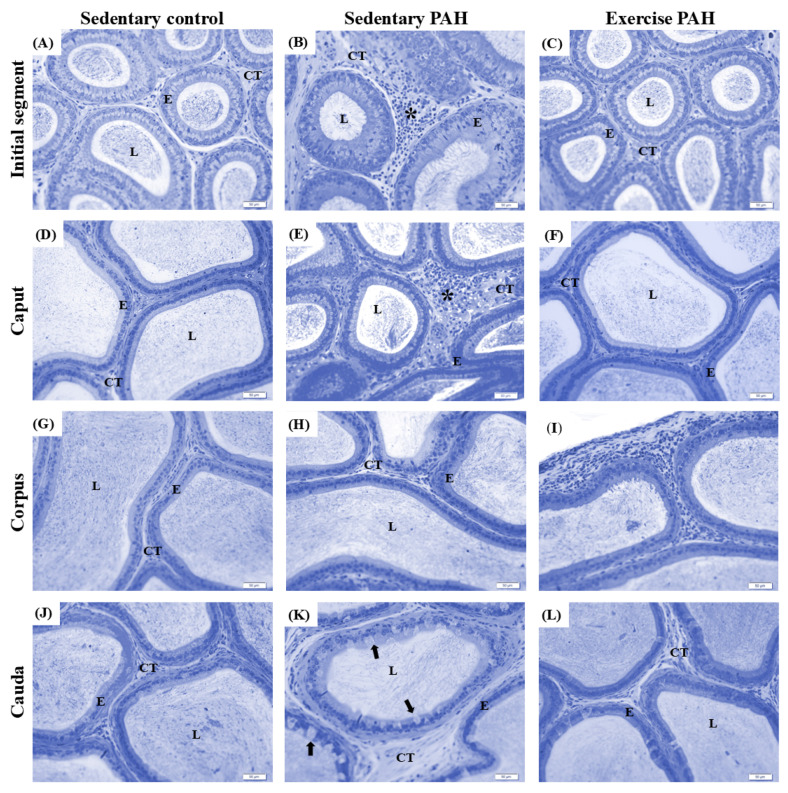
Photomicrographs of the four regions of the epididymis of Wistar rats. Namely, initial segment (**A**–**C**), caput (**D**–**F**), corpus (**G**–**I**), and cauda (**J**–**L**). Sedentary control group (**A**,**D**,**G**,**J**); sedentary PAH group (**B**,**E**,**H**,**K**); exercise PAH group (**C**,**F**,**I**,**L**). E, epididymal epithelium; L, lumen; CT: connective tissue; * focal points of inflammatory infiltrate. Black arrow: clear cell hyperplasia/hypertrophy. Toluidine blue. Scale bar: 50 µm (*n* = 5 animals/group).

Regarding epididymal histopathology, the tissue architecture was generally regular, with epithelium, lumen, and interstitium, without important changes in all duct regions for animals from the three experimental groups (Figure 3). However, focal points of inflammatory infiltrates were in the interstitium of the initial segment and caput of the epididymis of sedentary control and sedentary PAH animals (Figure 3B,E). In the cauda region, animals from both groups showed clear cell hyperplasia/hypertrophy, with greater regularity in animals from the sedentary PAH group (Figure 3K).

There was no change in the parameters of tubular and luminal diameters, height of the epithelium, and % of tubular compartment and lumen between control and sedentary PAH animals (*p* > 0.05; Table 2). Sedentary animals with PAH showed a decrease in the percentage of seminiferous epithelium and an increase in the proportion of tunica propria and intertubular compartments compared to sedentary control animals (*p* < 0.05; Table 2; Figure 2J,K).

Considering the components of the intertubule, sedentary animals with PAH did not show changes in the proportion and volume of connective tissue, lymphatic space, macrophages, and Leydig cells compared to animals in the sedentary control group (*p* > 0.05; Table 3). The volumetric proportion and volume of blood vessels between animals in the sedentary PAH group and those in the control group were increased (*p* < 0.05; Table 3).

Sedentary animals with PAH, when compared to control animals, did not show significant changes in the morphometric analyses of the Leydig cell (*p* > 0.05; Table S1).

**Figure 4:**
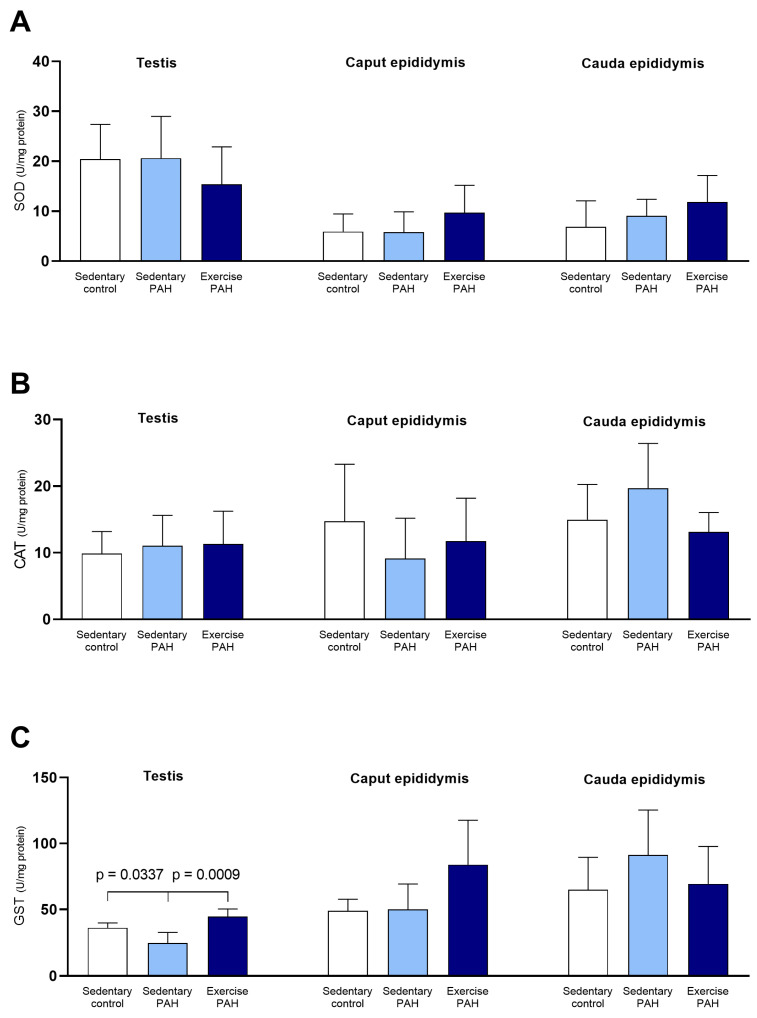
Effects of pulmonary arterial hypertension (PAH) and combined physical training on the activity of antioxidant enzymes in testis and epididymis (caput and cauda) of Wistar rats. One-way ANOVA followed by Tukey’s post hoc test (*n* = 5 animals/group). SOD = superoxide dismutase; CAT = catalase; GST = glutathione S-transferase. (**A**) SOD activity in the testis and epididymis (caput and cauda); (**B**) CAT activity in the testis and epididymis (caput and cauda); (**C**) GST activity in the testis and epididymis (caput and cauda).

The activities of testicular and epididymal CAT and SOD enzymes had no changes in PAH sedentary animals compared to control sedentary animals (*p* > 0.05; Figure 4A,B). There was a reduction in GST activity in the testis of animals in the sedentary PAH group compared to animals in the sedentary control group (*p* < 0.05; Figure 4C). On the other hand, epididymis GST activity in the caput and cauda regions did not change between animals (*p* > 0.05; Figure 4C).

**Figure 5:**
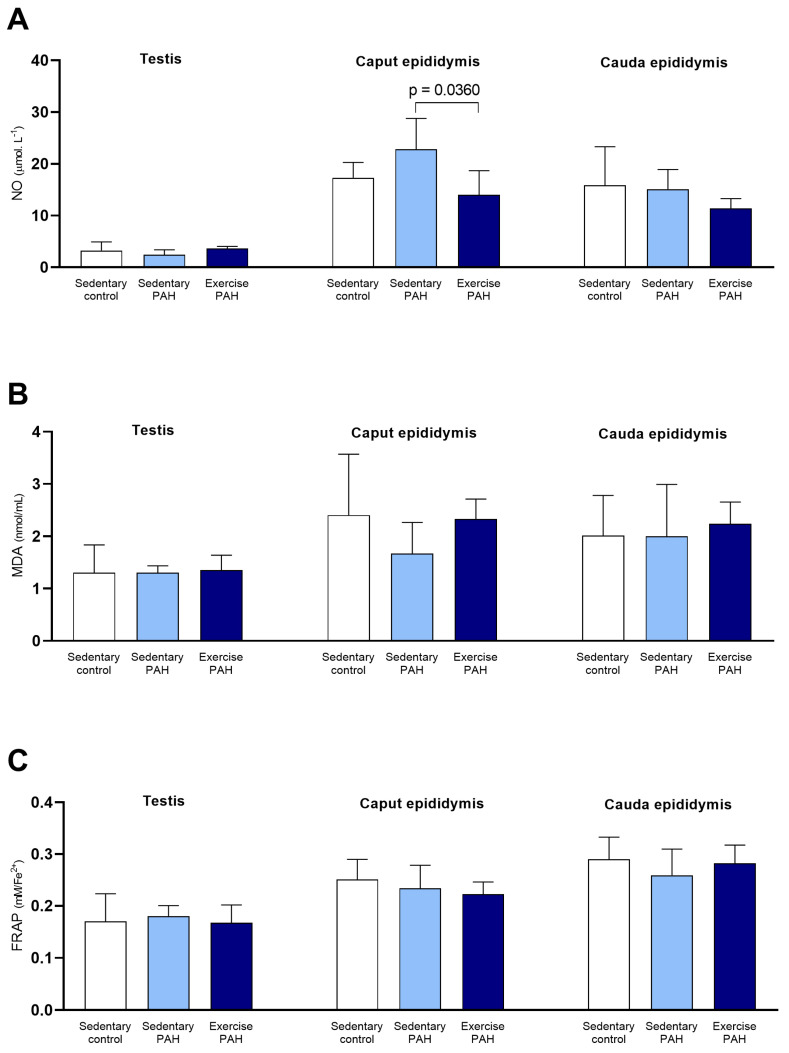
Effects of pulmonary arterial hypertension (PAH) and combined physical training on oxidative stress products and total antioxidant capacity in testis and epididymis (caput and cauda) of Wistar rats. One-way ANOVA followed by Tukey’s post hoc test (*n* = 5 animals/group). NO = nitric oxide; MDA = malondialdehyde; FRAP = Ferric-reducing antioxidant power. (**A**) NO level in the testis and epididymis (caput and cauda); (**B**) MDA concentration in the testis and epididymis (caput and cauda); (**C**) FRAP values in the testis and epididymis (caput and cauda).

Testicular and epididymal NO, MDA, and FRAP concentrations did not change between animals in the experimental groups (*p* > 0.05; Figure 5A–C).

The number of mature spermatids in the testis and per gram of testis, daily sperm production, the number of sperm in the caput/corpus and cauda of the epididymis and per gram of epididymis were reduced in sedentary PAH animals compared to sedentary control animals (*p* < 0.05, Table 4). The sperm transit time in the caput/corpus and cauda regions did not have significant changes between the animals in the experimental groups (*p* > 0.05, Table 4).

In the analysis of total sperm motility and morphology, there was no significant difference, and the percentage of normal and abnormal sperm (pathologies in the head, midpiece, and tail) was similar between animals in the sedentary control and sedentary PAH groups (*p* > 0.05, Table 4).

### 3.3. Effects of Combined Physical Training on Testicular and Epididymal Parameters of Hypertensive Rats

When comparing animals from the sedentary PAH and exercise PAH groups, there was no significant change in body (initial, final, and body weight gain), testicular, and epididymal biometric parameters (*p* > 0.05; Table 1).

It was shown that the animals from the sedentary PAH group, when compared to those animals in the exercise PAH group, increased the proportion of abnormal seminiferous tubules (*p* < 0.05; Figure 2E,F; Table 2).

As observed in sedentary animals and sedentary PAH groups, there were no significant changes in the epididymal architecture of exercise PAH animals (Figure 3C,F,I,L). Focal inflammatory infiltrates were observed in the interstitium of the initial segment in both sedentary PAH and exercise PAH animals. Notably, inflammatory infiltrates were found in the corpus only in the exercise PAH group. (Figure 3I). In the cauda region, animals from both groups showed clear cell hyperplasia/hypertrophy (Figure 3K).

There was no change in the parameters of tubular and luminal diameters, % tubular and intertubular compartments, and tunica propria among animals in the sedentary PAH and exercise PAH groups (*p* > 0.05; Table 2). Animals with PAH not subjected to combined physical training showed a decrease in height and the proportion of seminiferous epithelium, with an increase in the proportion of lumen compared with exercise PAH animals (*p* < 0.05; Table 2; Figure 2C,F,I). The proportion of lumen had a reduction in exercise PAH animals compared with sedentary control rats (*p* < 0.05; Table 2).

Considering the components of the intertubule, proportion and volume of connective tissue, lymphatic space, blood vessels, macrophages, and Leydig cells, no changes were found for the sedentary PAH and exercise PAH animals (*p* > 0.05; Table 3; Figure 2K,J). The proportion and volume of blood vessels increased among exercise PAH animals compared to sedentary control animals (*p* < 0.05; Table 3). Finally, no significant changes in the morphometric analyses of the Leydig cell were found between the animals of the experimental groups (*p* > 0.05; Table S1).

The activities of testicular and epididymal CAT and SOD enzymes did not change in sedentary PAH animals compared to exercise PAH animals (*p* > 0.05; Figure 4A,B). Sedentary animals with PAH had a decrease in GST activity compared to animals in the exercise PAH group (*p* < 0.001; Figure 4C). On the other hand, GST activity in the epididymis, in the caput and cauda regions, did not change between animals in the two experimental groups (*p* > 0.05; Figure 4C). Epididymal caput nitric oxide concentration increased between sedentary PAH animals and exercise PAH animals (*p* < 0.05; Figure 5A). There were no changes in NO concentration in the testis and the epididymis cauda between animals in the experimental groups (*p* > 0.05; Figure 5A). The values of MDA and FRAP, testicular and epididymal, did not show changes between the animals in the experimental groups (*p* > 0.05; Figure 5B,C).

The number of mature spermatids in the testis and per gram of testis and daily sperm production did not change significantly in the sedentary PAH animals compared to the exercise PAH animals (*p* > 0.05, Table 4). The number of sperm in the caput/corpus and cauda of the epididymis and per gram of epididymis decreased in sedentary PAH animals compared to animals that practiced combined physical training and were hypertensive (*p* < 0.05, Table 4). The sperm transit time in the caput/corpus and cauda regions did not have significant changes between the animals in the experimental groups (*p* > 0.05, Table 4). The number of mature spermatids per gram of testis and the number of sperm in the epididymis cauda per gram of the organ decreased in exercise PAH animals compared with sedentary control animals (*p* < 0.05, Table 4).

There was no change in sperm motility and morphology between sedentary PAH animals and those in the exercise PAH group (*p* > 0.05, Table 4).

## 4. Discussion

This study investigates the effects of PAH and combined physical training on testicular and epididymal morphology and physiology and sperm parameters in adult Wistar rats with PAH. The results evidenced that PAH compromises testicular histomorphometry, reduces serum testosterone levels, and decreases sperm count in the testis and epididymis. They corroborate previous reports on the detrimental effects of monocrotaline-induced PAH [25,26]. Although combined physical training showed protective effects on several testicular parameters, its benefits for the epididymis were limited.

### 4.1. Impact of PAH on Testicular and Epididymal Parameters

Intraperitoneal injection of monocrotaline successfully induced PAH, as evidenced by echocardiography, a widely used diagnostic and prognostic tool in patients with this condition [61]. PAH resulted in impeded growth of body weight and epididymal weight—the latter, possibly at the expense of detrimental testosterone levels. These findings align with previous studies reporting body weight loss in monocrotaline-induced PAH models [62,63]. The observed reduction in epididymal weight further highlights the systemic impacts of PAH on male reproductive health. Biometric parameters are critical indicators of overall animal health, suggesting that PAH negatively impacts this status [64]. The reduction in absolute epididymal weight in sedentary hypertensive animals reflects organ dysfunction [65]. Testosterone is critical for developing and maintaining reproductive organs, essential for spermatogenesis and epididymal function [66,67]. Herein, the maintenance of testicular weight in hypertensive animals probably occurred because of increased blood vessels in the intertubular compartment.

Histological analysis revealed a significant height in the percentage of abnormal seminiferous tubules in PAH animals. These tubules exhibited signs of disorganization, such as detachment of germ cells and cellular debris in the lumen. Sertoli cell dysfunction may explain these abnormalities. Sertoli cells are crucial for maintaining the blood-testicular barrier and supporting germ cell development, and their impairment may compromise spermatogenesis [66,68]. In a recent study, Guimarães-Ervilha et al. [25] revealed alterations in the autophagic activity of seminiferous epithelial cells of animals with PAH, which may compromise the proper functioning and organization of the seminiferous tubules. Furthermore, hormonal problems, such as alterations in the connection of the luteinizing hormone (LH) and follicle-stimulating hormone (FSH), may impair the integrity of the seminiferous epithelium. Men with PAH present alterations in the concentration of these essential hormones [69].

Epididymal histopathology showed the presence of focal inflammatory infiltrates, particularly in the initial segment and caput regions, and clear cell hyperplasia/hypertrophy in the cauda region. These alterations suggest a PAH-induced inflammatory response. Indeed, inflammation appears to mediate the pathogenesis of hypertension, with increased vascular permeability through the release of potent mediators, such as cytokines (interleukins, growth factors) and ROS (O^2−^, H_2_O_2_) [70,71]. The epididymis, more susceptible to immune-mediated damage than the testis, appears disproportionately affected by PAH [72]. Epididymal inflammatory processes are a relevant factor affecting male fertility [73]. Herein, clear cell hyperplasia/hypertrophy in the cauda region of animals from both PAH groups indicates a breakdown of epithelium homeostasis. Clear cells become abnormally large and filled with lysosomes (involved in endocytosis), potentially disrupting the regular epididymal environment required for sperm maturation and storage [43,74].

In the testis, the reduced volume and proportion of functional components of the tubular compartment, including the low epithelium percentage, indicated a low efficiency in sperm production in hypertensive animals [75]. Data obtained from seminiferous epithelium is a more effective way to assess sperm production conditions since the epithelium is a dynamic element in the seminiferous tubule, which undergoes variations according to the seminiferous cycle [76]. Therefore, these findings suggest that sperm production was compromised under hypertensive conditions, reflected in the number of sperm counted in the epididymis. In contrast, the portion of the testicular parenchyma corresponding to the intertubular space had a compensatory increase in sedentary PAH animals. This fact is in line, for example, with the increase in the volumetric proportion and volume of blood vessels in the testicular intertubule. Considering the nature of PAH, it seems plausible that this increase in vascular parameters was due to blood vessel remodeling, one of the histopathological alterations observed in this disease [77,78].

PAH could not promote changes in the morphometry of Leydig cells, possibly due to the duration of the experiment. Leydig cells can suffer external and internal disturbances, mainly due to oxidative stress, which can compromise their functions [79]. Although oxidative stress is an important mechanism in the pathophysiology of PAH [6,80], this study did not observe oxidative imbalance. Interestingly, only GST activity was reduced by PAH, possibly due to the reduction of the substrate GSH, which can be used in excess in detoxification processes. These effects were observed in the testis and epididymis of animals with systemic arterial hypertension induced by N(G)-nitro-L-arginine methyl ester (L-NAME) [81]. Therefore, the mechanisms by which the disease modulates the activity of this enzyme need to be studied. These findings suggest that PAH may selectively impair specific antioxidant pathways without causing overt oxidative imbalance.

Daily sperm production and testicular spermatid counts decreased in sedentary PAH animals as a consequence of the histological alterations in the testis. These two parameters are important indicators of male fertility potential, as spermatids are germline cells involved in spermiogenesis and daily sperm production [82]. Low sperm production implies a low number of sperm cells reaching the epididymal regions (caput, corpus, and cauda), which was confirmed here. The time spermatozoa spend transiting within the entire epididymal duct to undergo the maturation process is known as sperm transit time [82]. In rats, this journey takes about eight days [56,74]. Therefore, maintaining sperm transit time is crucial for preserving sperm cells with normal morphology.

Overall, oxidative stress and inflammation act synergistically in the pathogenesis of PAH. Together, they increase blood pressure and provoke right ventricular remodeling, compromising vascular perfusion and blood flow to peripheral organs, including the testis [83,84]. Testicular vascularization is essential for thermoregulation and transportation of nutrients, oxygen, and hormones and is highly vulnerable to hemodynamic changes and oxidative stress [85]. Cardiovascular diseases, such as systemic arterial hypertension, reduce testicular microcirculation and increase oxidative stress, impairing gonadal physiology [22], whereas the activation of the inflammatory cascade can compromise spermatogenesis [73]. One of the main mechanisms associated with redox imbalance is the downregulation of nuclear factor erythroid 2-related factor (Nrf2), which favors inflammatory pathways and leads to testicular damage and low sperm production in hypertensive rats [86,87]. Also, PAH negatively impacts testosterone production [25], and its low levels may increase the risk of adverse cardiovascular events [88]. Thus, androgens influence blood pressure regulation [21], modulating endothelial function and affecting steroidogenesis [88].

### 4.2. Protective Role of Combined Physical Training

Animals with PAH that underwent combined physical training improved in cardiac parameters, consistent with prior studies that evaluated PAH using the monocrotaline induction model and demonstrated the cardiopulmonary benefits of physical exercise in rats [18,34,35,38,89]. Combined exercise training partially mitigated the adverse effects of PAH on testicular parameters. Exercise increased the proportion of normal seminiferous tubules, increased the height of the seminiferous epithelium, and reduced the lumen ratio. These findings suggest that combined resistance and aerobic exercise training help preserve the testicular architecture and spermatogenesis, probably through its anti-inflammatory effects and antioxidant pathway enhancement [8,10]. Improvements in sperm counts in the caput and cauda epididymis also support the protective role of exercise in maintaining reproductive function.

The benefits of exercise training for the epididymis, however, were limited. Although some parameters, such as sperm count, showed improvement, histological changes in the epididymis persisted, including inflammatory infiltrates and clear cell hyperplasia. These findings may indicate that the short duration of the intervention was insufficient to protect the epididymis from the progression of PAH. Future studies with more extended training periods may provide more insights into the time-dependent effects of exercise training on the epididymis.

Exercise is known to increase the bioavailability of NO, which plays a critical role in vascular and reproductive health [16,90]. During exercise training, there is an increase in shear stress that increases the expression of the enzyme NO synthase, which produces endogenous NO [16]. Although we did not observe changes in the activities of antioxidant enzymes and oxidative markers in the epididymis, nitric oxide levels were elevated in the caput of the epididymis in animals from the sedentary PAH group. Studies show that along the epididymal duct, NO synthases are mainly present in the caput region [91,92]. In this context, although the role of epididymal NO in sperm maturation is not fully elucidated, it is known that at higher concentrations, the acquisition of motility and viability are impaired. On the other hand, NO (in endogenous concentrations) appears to be necessary for adequate sperm motility and viability [92,93]. Thus, exercise may be necessary for sperm maintenance so sperm acquires its functional maturity in the proximal segments before being stored in a quiescent state in the cauda epididymis [94].

It is known that physical training is important for improving blood flow and pressure [95,96], factors compromised by PAH. This regular practice can enhance endothelial cell functions, reducing oxidative and inflammatory damage [95,96,97] since it promotes an increase in NO bioavailability and the activation of factors that increase enzymatic antioxidant defense and the formation of anti-inflammatory cytokines [96,98]. Thus, physical exercise may have protected testicular functions, such as sperm production, through improved vascular health and reduced oxidative imbalance.

### 4.3. Limitations

The relatively short intervention period used here resulted from the short survival period of PAH-induced animals. Hence, it limited the full protective potential of combined exercise training, particularly for the epididymis. In addition to the reproductive system, where data are still lacking in the literature, combined exercise training improves other parameters affected by PAH [99,100]. Combined exercise training with aerobic and resistance inspiratory muscle training for 8 weeks was safe and induced improvements in muscle strength and maximal oxygen consumption in patients with PAH [101]. In another study, during 15 weeks of training, patients showed improvements in respiratory muscle strength and exercise capacity [102]. The combined physical training improved pulmonary and cardiac parameters in hypertensive animals induced by monocrotaline and was crucial in preventing dysfunctional aspects of the disease [34,35].

Animal models provide relevant information about PAH development and maintenance pathways. However, there is still no ideal preclinical model for human PAH [103], which may influence the translation of the findings to clinical forms of the disease in humans. It is still necessary to develop new experimental models that better simulate hypertension conditions, possibly using more than one animal model of PAH to generate and improve findings for treating this disease [104].

Our study does not compare the effects of combined exercise training with other therapeutic approaches, such as pharmacological treatments. This comparison with other therapies would provide more robust clues about the efficacy of exercise training as a treatment for reproductive dysfunction caused by PAH. Moreover, the duration and intensity of combined physical training may have limited its effectiveness in preventing the effects of PAH on the epididymis. Physical exercise has only recently been included in the treatment options for pulmonary hypertension. Therefore, evidence still needs to be better substantiated to ensure the safety and efficacy of the adopted strategies [84].

### 4.4. Clinical Implications and Future Directions

The findings highlight the potential of physical exercise, specifically combined physical training, as a non-pharmacological strategy to mitigate reproductive damage induced by cardiovascular diseases such as PAH. Although the benefits were more evident in the testis, the partial protection observed in the epididymis evidences the need for comprehensive and combined therapeutic approaches. For example, the association of physical exercise with antioxidant and/or anti-inflammatory compounds may improve the protective effect of exercise against changes caused by PAH progression. These results contribute to a better understanding of the interaction between cardiovascular diseases, physical exercise, and male reproductive disorders, paving the way for translational research.

Understanding the potential mechanisms underlying PAH effects may also support the development of targeted therapeutic strategies, focusing on the consequences of hormonal and inflammatory imbalances and contributing to ameliorating testicular and epididymal dysfunction. The physiological effects of steroid hormones seem more complex and less elucidated [104,105,106]. In addition, pharmacological treatment and the implementation of physical exercise practice provide lower costs and important health benefits for patients [1,107]. Further investigation is needed to explore whether different training modalities and/or the combination with other interventions may have a more pronounced effect on the epididymis. Therefore, it remains essential to investigate various forms of physical training interventions and develop a deeper understanding of their efficacy on PAH molecular and physiological factors [84,108].

## 5. Conclusions

This study highlights the interplay between PAH and male reproductive function and provides valuable information for understanding the effects of this disease on the testis and epididymis. Our results evidenced testicular and post-testicular alterations caused by PAH, culminating in low sperm production. Indeed, hypertensive animals showed low body weight, a low percentage of normal seminiferous tubules, and altered seminiferous epithelium proportion. Collectively, these findings reflected the sperm production impairment. Combined physical training promoted significant changes in testicular morphometry and sperm count in the epididymis. However, under our experimental conditions, the exercise did not demonstrate effectiveness as a therapeutic approach to minimize or prevent the effects of PAH on the epididymis. Therefore, exercise training may be a promising complementary strategy to improve hypertensive patients’ quality of life and reproductive functions. Further studies are needed to elucidate the molecular mechanisms of PAH in affecting male reproductive organs. Also, alternative therapeutic strategies, including longer interventions or combination with drugs (e.g., antioxidants and anti-inflammatories), should be explored to mitigate the effects of PAH, especially in the epididymis.

## Figures and Tables

**Table 1:** Echocardiography, body weight, biometry of testicular and epididymal, and serum testosterone concentration of healthy Wistar rats (sedentary control) or with pulmonary arterial hypertension (PAH) induced by monocrotaline, submitted or not to combined physical training.

Parameters	Sedentary Control	Sedentary PAH	Exercise PAH
TAPSE	2.50 ± 0.37	1.43 ± 0.10 ****^###^	2.30 ± 0.24
Acceleration/ejection time ratio (TA/TE)	0.51 ± 0.03	0.38 ± 0.03 ****^####^	0.52 ± 0.04
Initial body weight (g)	197.40 ± 14.24	192.80 ± 10.23	207.40 ± 11.26
Final body weight (g)	306.00 ± 15.76	267.40 ± 19.57 *	274.00 ± 29.81
Body weight gain (g)	108.60 ± 26.27	74.60 ± 18.41	66.60 ± 35.66
Testis (g)	1.29 ± 0.16	1.16 ± 0.10	1.20 ± 0.15
Testis (g/100 g)	0.84 ± 0.11	0.87 ± 0.09	0.87 ± 0.06
Epididymis (g)	0.46 ± 0.04	0.39 ± 0.04 *	0.40 ± 0.04
Epididymis (g/100 g)	0.31 ± 0.04	0.29 ± 0.03	0.29 ± 0.03

Values expressed as mean ± S.D.M. * Significant difference vs. sedentary control animals. ^#^ Significant difference vs. exercise PAH animals. One-way ANOVA followed by Tukey’s post hoc test (*n* = 6 animals/group). * *p* < 0.05; ^###^ *p* < 0.01; ****^,####^ *p* < 0.0001.

**Table 2:** Testicular histomorphometric and stereological parameters of healthy Wistar rats (sedentary control) or with pulmonary arterial hypertension (PAH) induced by monocrotaline, submitted or not to combined physical training.

Parameters	Sedentary Control	Sedentary PAH	Exercise PAH
*Histopathology of seminiferous tubules*			
Abnormal tubules (%)	6.20 ± 0.76	9.70 ± 2.91 *^#^	6.10 ± 1.56
*Testicular morphometry*			
Tubular diameter (μm)	396.70 ± 7.52	392.40 ± 13.18	393.70 ± 17.70
Luminal diameter (μm)	185.90 ± 7.98	196.00 ± 13.93	186.30 ± 10.62
Epithelium height (μm)	104.00 ± 4.59	98.21 ± 1.52 ^#^	104.60 ± 4.11
*Volumetric proportion*			
Tubular compartment (%)	88.43 ± 2.69	84.55 ± 1.94	87.87 ± 2.43
Seminiferous epithelium (%)	66.88 ± 2.50	62.24 ± 2.87 *^#^	68.91 ± 2.00
Tunica propria (%)	4.84 ± 0.53	6.43 ± 1.04 *	6.12 ± 0.68
Lumen (%)	16.71 ± 1.80	15.88 ± 2.22 ^#^	12.83 ± 1.39 *
Intertubular compartment (%)	11.57 ± 2.69	16.03 ± 1.65 *	12.13 ± 2.43

Values expressed as mean ± S.D.M. * Significant difference vs. sedentary control animals. ^#^ Significant difference vs. exercise PAH animals. One-way ANOVA followed by Tukey’s post hoc test (*n* = 5 animals/group). *^,#^ *p* < 0.05.

**Table 3:** Histomorphometric and stereological parameters—volumetric proportion and volume of components of the testicular intertubule of healthy Wistar rats (sedentary control) or with pulmonary arterial hypertension (PAH) induced by monocrotaline, submitted or not to combined physical training.

Parameters	Sedentary Control	Sedentary PAH	Exercise PAH
*Volumetric proportion*			
Connective tissue (%)	0.44 ± 0.18	0.64 ± 0.20	0.36 ± 0.17
Lymphatic space (%)	3.90 ± 0.80	6.09 ± 2.08	3.78 ± 0.89
Blood vessel (%)	0.72 ± 0.20	1.49 ± 0.25 *	1.67 ± 0.79 *
Macrophages (%)	0.06 ± 0.03	0.06 ± 0.08	0.01 ± 0.02
Leydig cell (%)	6.40 ± 2.32	7.17 ± 0.57	6.30 ± 1.00
*Volume*			
Connective tissue (mL)	0.007 ± 0.003	0.012 ± 0.004	0.007 ± 0.003
Lymphatic space (mL)	0.068 ± 0.017	0.120 ± 0.050	0.071 ± 0.021
Blood vessel (mL)	0.012 ± 0.002	0.028 ± 0.007 *	0.032 ± 0.019 *
Macrophages (mL)	0.001 ± 0.001	0.001 ± 0.002	0.0002 ± 0.0004
Leydig cell (mL)	0.110 ± 0.037	0.135 ± 0.018	0.117 ± 0.025

Values expressed as mean ± S.D.M. * Significant difference vs. sedentary control animals. ^#^ Significant difference vs. exercise PAH animals. One-way ANOVA followed by Tukey’s post hoc test (*n* = 5 animals/group). * *p* < 0.05.

**Table 4:** Sperm parameters (morphology and motility) and sperm count of healthy Wistar rats (sedentary control) or with pulmonary arterial hypertension (PAH) induced by monocrotaline, submitted or not to combined physical training.

Parameters	Sedentary Control	Sedentary PAH	Exercise PAH
*Sperm count*			
Number of spermatids (×10^6^/testis)	172.00 ± 20.55	105.90 ± 20.18 *	131.80 ± 29.84
Number of spermatids (×10^6^/g testis)	134.00 ± 13.19	90.11 ± 23.15 *	96.21 ± 14.83 *
Daily sperm production (×10^6^/testis/day)	28.20 ± 3.37	17.35 ± 3.31 *	21.61 ± 4.89
Number of sperm in the caput/corpus of the epididymis (×10^6^/organ)	85.48 ± 9.02	50.99 ± 9.95 *^#^	83.59 ± 20.05
Number of sperm in the caput/corpus of the epididymis (×10^6^/g organ)	402.30 ± 47.08	287.80 ± 36.61 *^#^	417.00 ± 93.21
Sperm transit time in the caput/corpus of the epididymis (days)	3.05 ± 0.33	3.07 ± 1.03	3.87 ± 0.30
Number of sperm in the epididymis cauda (×10^6^/organ)	120.40 ± 22.21	74.28 ± 14.65 *^#^	124.40 ± 38.78
Number of sperm in the epididymis cauda (organ ×10^6^/g)	926.50 ± 47.98	622.50 ± 64.98 ***^#^	792.50 ± 109.60 *
Sperm transit time in the cauda of the epididymis (days)	4.33 ± 0.98	4.54 ± 1.76	5.12 ± 1.30
*Sperm parameters*			
Sperm motility (%)	81.40 ± 4.72	74.00 ± 4.18	74.60 ± 5.08
Normal morphology (%)	98.40 ± 0.82	98.20 ± 0.76	99.13 ± 0.75
Abnormal morphology (%)	1.60 ± 0.82	1.80 ± 0.76	0.88 ± 0.75

Values expressed as mean ± S.D.M. * Significant difference vs. sedentary control animals. ^#^ Significant difference vs. exercise PAH animals. One-way ANOVA followed by Tukey’s post hoc test (*n* = 5 animals/group). *^,#^ *p* < 0.05; *** *p* < 0.001.

## Data Availability

The original contributions presented in this study are included in the article; further inquiries can be directed to the corresponding author.

## Supplementary Materials

The following supporting information can be downloaded at: https://www.mdpi.com/article/10.3390/biomedicines13020410/s1, Table S1: Leydig cell stereology from healthy Wistar rats (sedentary control) or with pulmonary arterial hypertension (PAH) induced by monocrotaline, submitted or not to combined physical training.
