# Supplementary material for: Pulmonary Arterial Hypertension-Induced Reproductive Damage: Effects of Combined Physical Training on Testicular and Epididymal Parameters in Rats
Source: Biomedicines. 2025 Feb 8;13(2):410. doi: 10.3390/biomedicines13020410 (PMC11853577; doi:10.3390/biomedicines13020410)
Supplement: Supplementary file 1 [file biomedicines-13-00410-s001.zip › biomedicines-3447194-supplementary.pdf]

Table S1. Leydig cell stereology from healthy Wistar rats (sedentary control) or with pulmonary arterial hypertension (PAH) induced by monocrotaline, submitted or not to combined physical training.

Parameters	Sedentary control	Sedentary PAH	Exercise PAH
Nuclear diameter (μm)	6.76 ± 0.29	6.74 ± 0.19	6.78 ± 0.06
Nuclear percentage (%)	0.85 ± 0.34	1.05 ± 0.27	0.90 ± 0.12
Cytoplasmic percentage (%)	5.55 ± 1.99	6.12 ± 0.52	5.39 ± 0.99
Nuclear volume (μm^3)	162.10 ± 19.79	160.50 ± 13.75	163.30 ± 4.49
Cytoplasmic volume (μm^3)	1,079 ± 194.00	997.70 ± 296.60	987.40 ± 240.30
Cell volume (μm^3)	1,242 ± 210.80	1,158 ± 300.80	1,151 ± 239.70
Number of cells/testis ($\times 10^6$)	87.94 ± 27.59	105.80 ± 20.41	102.10 ± 6.62

Values expressed as mean ± S.D.M. (n = 5 animals/group).
